# EHMTI-0191. A novel approach for the treatment of cluster headache – onabotulinumtoxina block of the sphenopalatine ganglion

**DOI:** 10.1186/1129-2377-15-S1-E4

**Published:** 2014-09-18

**Authors:** D Bratbak, S Nordgård, L Stovner, M Linde, E Tronvik

**Affiliations:** 1 Department of Neuroscience, Norwegian University of Science and Technology, Trondheim, Norway

## Background

Blockade of the sphenopalatine ganglion with OnabotulinumtoxinA injections (SphenoBlock) represents a novel approach for treating intractable chronic cluster headache (iCCH).

## Aim

The aim of this pilot study was to explore the safety aspects and therapeutic potential of SphenoBlock.

## Method

After signing written confirmed consent ten patients with iCCH were injected with 25 U (n=5) or 50 U (n=5) onabotulinumtoxinA towards the sphenopalatine ganglion in an observational study, approved by the Institutional Review Board, with 6 months follow-up. The procedure was performed with a novel image-guided technique. The primary endpoint was to evaluate safety of the procedure, but change in attack frequency from baseline to week 4, 12 and 24 was also registered.

## Results

**Figure 1:**
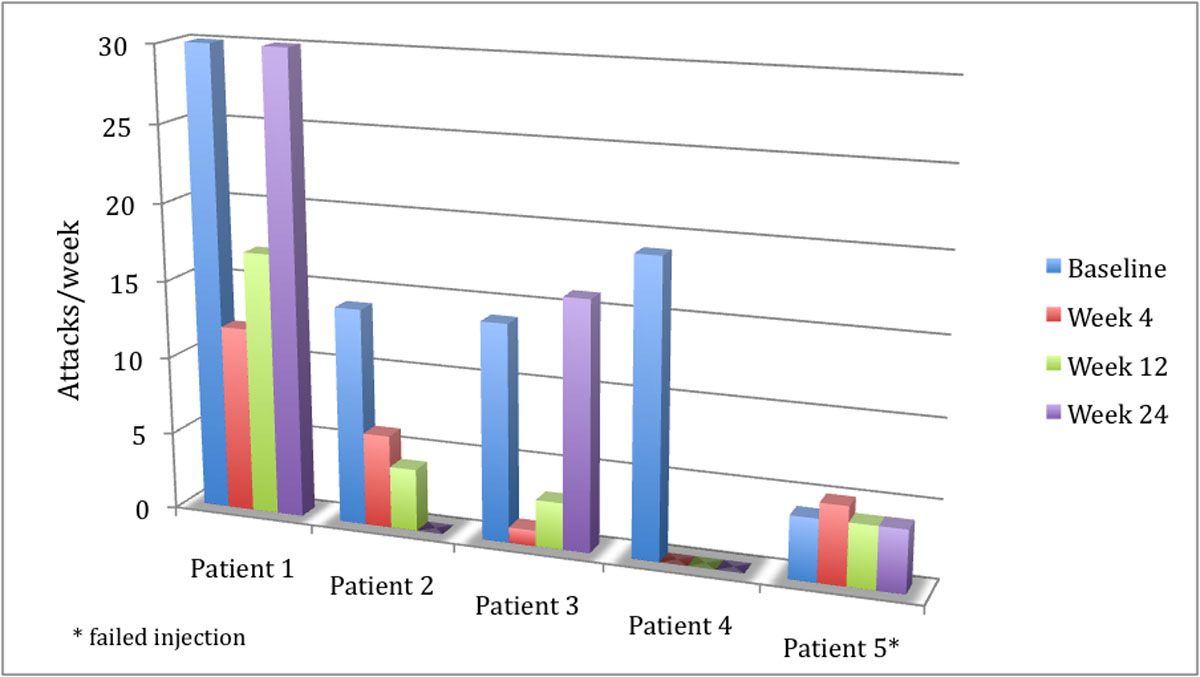


Data for the first 5 patients are presented. One patient experienced intermittent ipsilateral visual deficits lasting 4 weeks. Patient number 5 was a failed injection. Four patients were defined as frequency responders (>50% reduction from baseline) in week 4, 3 patients responded in week 12, and 2 patients in week 24 (Figure 1). Complete study data will be presented at the meeting.

## Conclusion

SphenoBlock in iCCH shows promising preliminary results and give reasons for cautious optimism for further studies on this low-cost alternative treatment of iCCH.

